# Supplementary material for: Intrinsic and synaptic determinants of receptive field plasticity in Purkinje cells of the mouse cerebellum
Source: Nat Commun. 2024 May 31;15:4645. doi: 10.1038/s41467-024-48373-3 (PMC11143328; doi:10.1038/s41467-024-48373-3)
Supplement: Supplementary file 1 — Supplementary Information [file 41467_2024_48373_MOESM1_ESM.pdf]

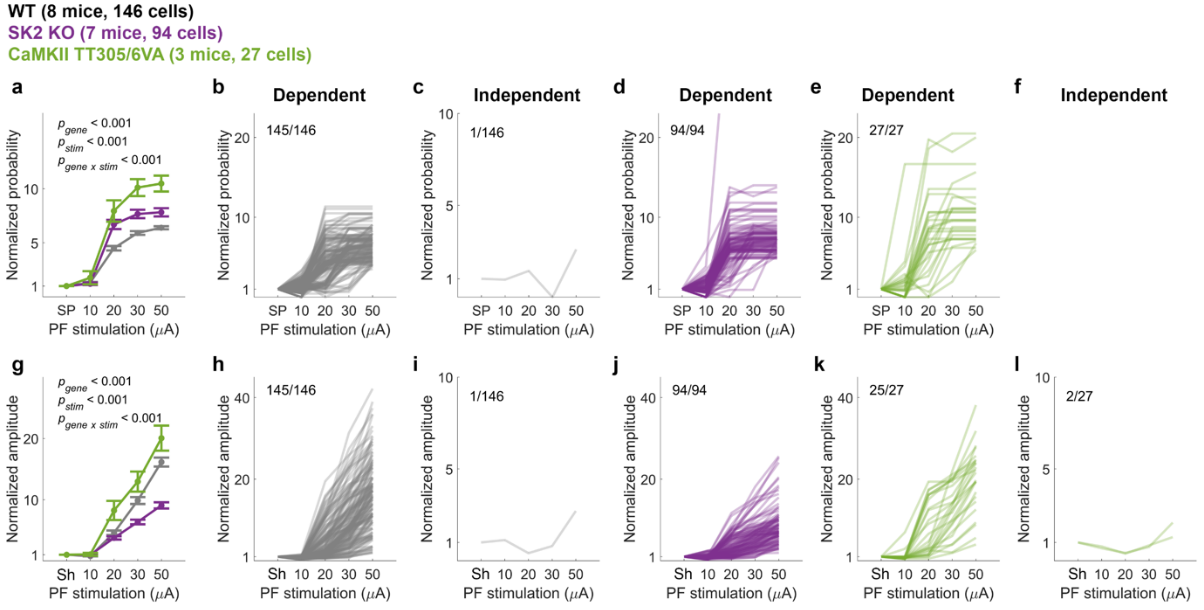

Supplementary Fig. 1 PCs show both linear stimulus intensity-dependent and flat stimulus intensity-independent responses to PF stimulation.

a. Calcium-event probability (mean ± SEM) as depicted in Fig. 2c normalized by the spontaneous values of individual cells. (Two-way ANOVA: $F_{stim}[4,1320] = 328.39$, $p < 0.001$; $F_{gene}[2,1320] = 65.61$, $p < 0.001$; $F_{stim\ x\ gene}[8,1320] = 10.58$, $p < 0.001$, $n = 1335$).

b-f. Stimulus intensity-dependent (**b, d and e**) and stimulus intensity-independent cells (**c**) categorized according to the correlation coefficient of normalized probability. The labels on top of the plots indicate the number of designated cells out of the total number. The data are derived from the data presented in **a**, but only include the trials that were tested under all four different stimulus conditions.

g. Calcium-event amplitude (mean ± SEM) as depicted in Fig. 2d normalized by the maximum values of individual cells during shuffled 200ms time windows. (Two-way ANOVA: $F_{stim}[4,1320] = 237.35$, $p < 0.001$; $F_{gene}[2,1320] = 53.17$, $p < 0.001$; $F_{stim\ x\ gene}[8, 1320] = 15.43$, $p < 0.001$, $n = 1335$).

h-l. Stimulus intensity-dependent (**h, j and k**) and stimulus intensity-independent cells (**h, j and l**) categorized according to the correlation coefficient of normalized probability. The labels on top of the plots indicate the number of designated cells out of the total number. The data are derived from the data presented in **f**, but only include the trials that were tested under all four different stimulus conditions.

Only cells that were tested with all four stimulus intensities were included in the categorization, which include wild type (146 cells), SK2 KO (94 cells), CaMKII TT305/6VA (27 cells).

SP, spontaneous calcium events. Sh, shuffled traces.

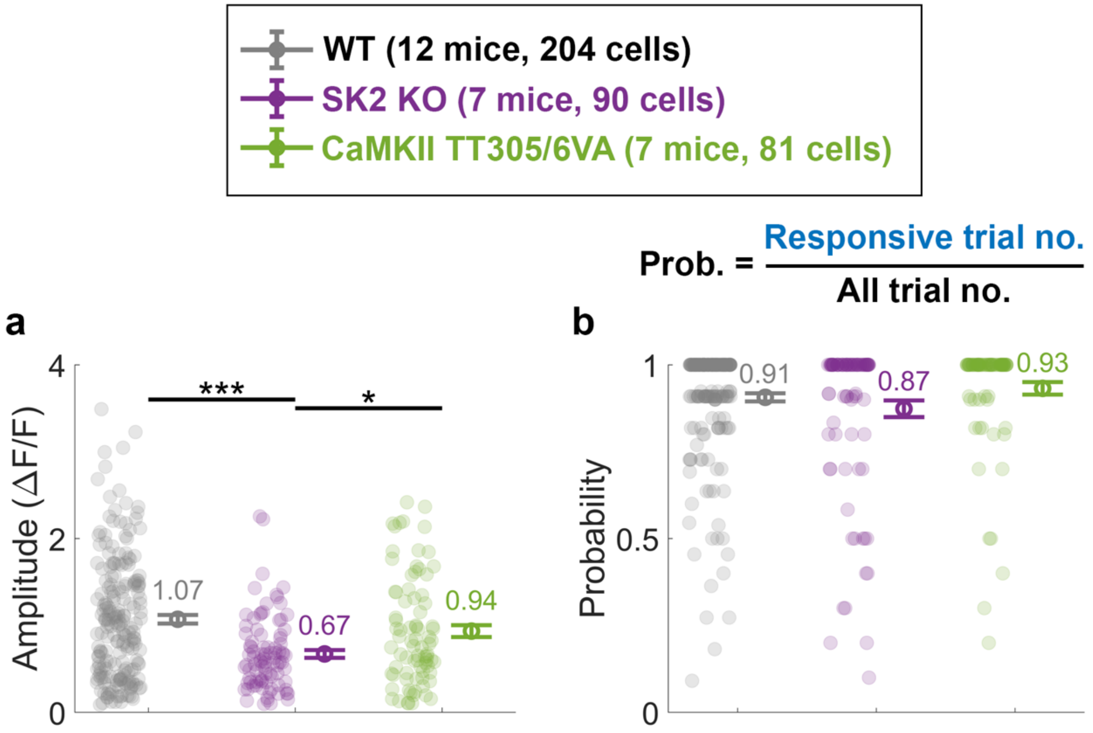

Supplementary Fig. 2 Amplitude and probability of pre-tetanus calcium events in response to PF stimulation.

a. Mean ± SEM of the pre-tetanus amplitudes of calcium transients for responsive trials shown in Fig. 3c-3d. (One-way ANOVA: $F[2,372] = 12.61$, $p <0.001$, $n = 375$).

b. Mean ± SEM of the pre-tetanus probability of detecting a calcium event within the defined time window of 0-200ms. (One-way ANOVA: $F[2,372] = 2.28$, $p = 0.104$, $n = 375$).

Numbers indicate the group mean values. Asterisks denote the significance levels of post hoc comparisons using Tukey's HSD (*, $p < 0.05$; **, $p < 0.01$; ***, $p < 0.001$).

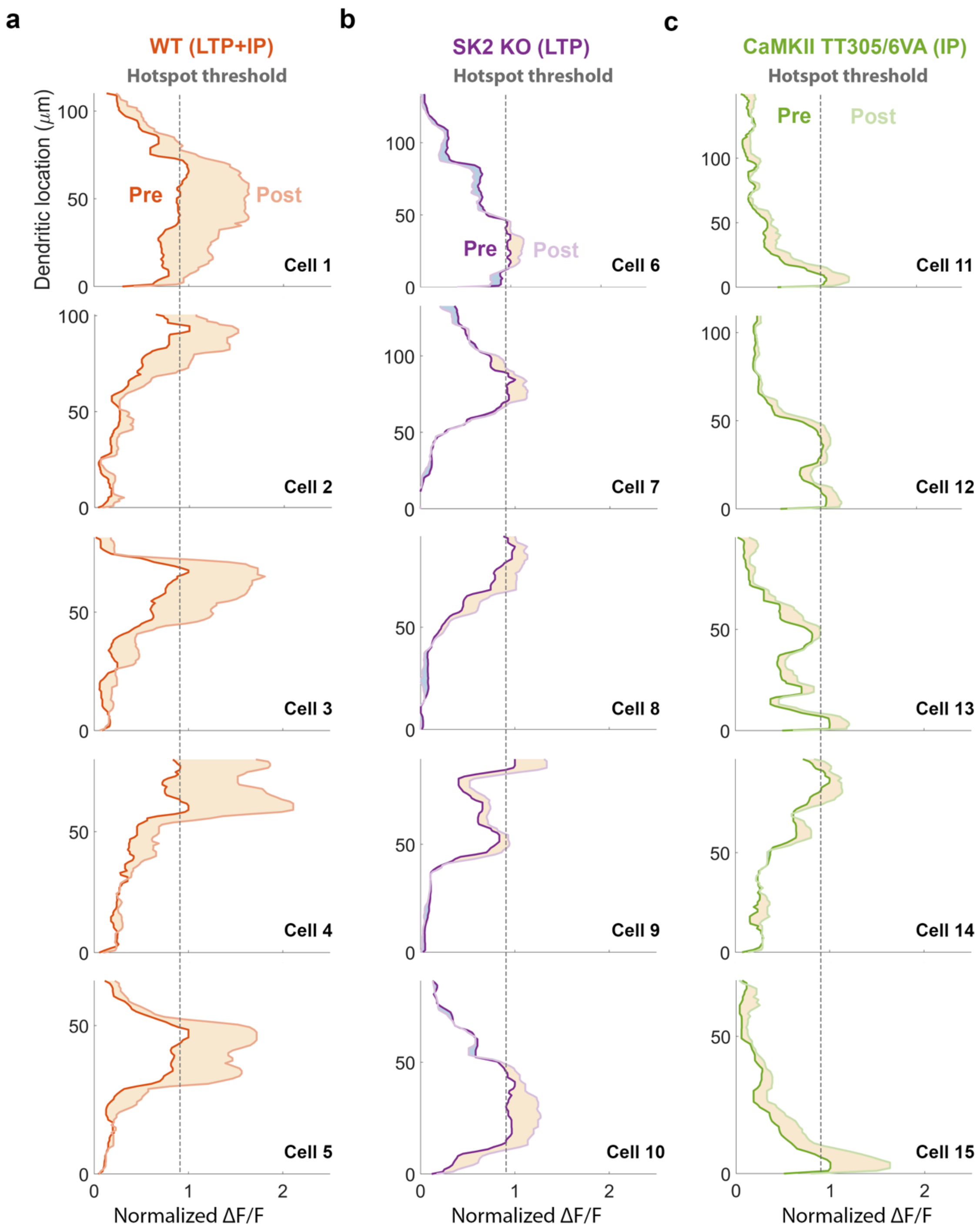

Supplementary Fig. 3 Representative spatial patterns of PF calcium response during pre-tetanus and early post-tetanus periods.

a-c. Representative linescan fluorescence signals of 5 individual PCs per genotype in addition to those shown in Fig. 4e-4g. The dark and light traces represent pre- and (early) post-tetanus data, respectively. The dashed lines indicate hotspot area (pixels with >0.9 normalized pre-tetanus ΔF/F). LTP, long-term potentiation. IP, intrinsic plasticity.

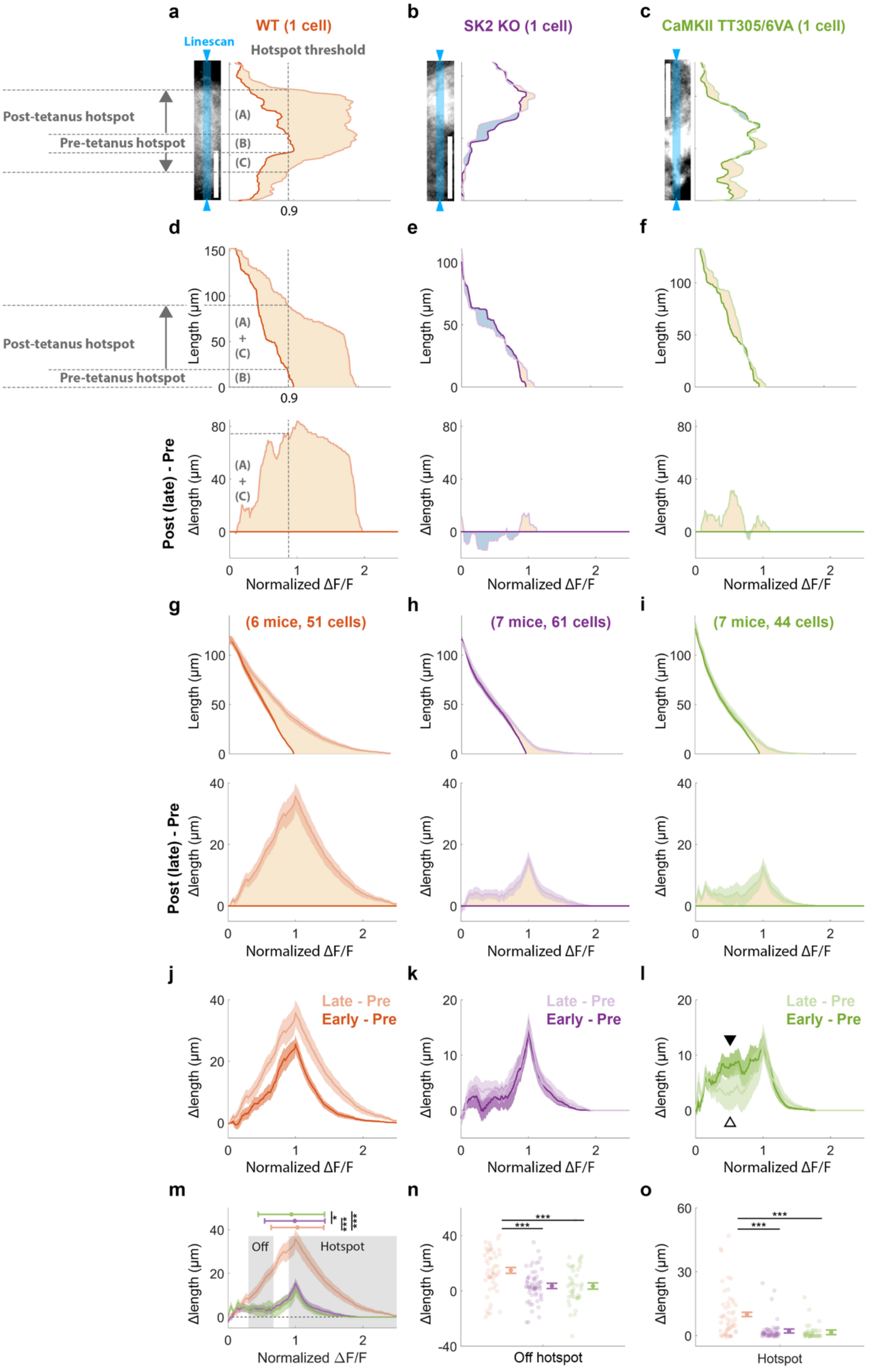

a
WT (1 cell)
Linescan
Hotspot threshold
Post-tetanus hotspot
Pre-tetanus hotspot
(A)
(B)
(C)
0.9
b
SK2 KO (1 cell)
c
CaMKII TT305/6VA (1 cell)
d
e
f
Length (μm)
(A)
+
(C)
(B)
0.9
Post (late) - Pre
Δlength (μm)
Normalized ΔF/F
g
(6 mice, 51 cells)
h
(7 mice, 61 cells)
i
(7 mice, 44 cells)
j
k
l
Late - Pre
Early - Pre
m
Off
Hotspot
*
***
***
n
***
***
Off hotspot
o
***
***
Hotspot

Supplementary Fig. 4 PF calcium spatial patterns are similar across genotypes between early and late post-tetanus periods but show some transience in mutant animals.

a-c. Representative two-photon images of individual PCs from each genotype are shown in the left panels, with corresponding linescan fluorescence signals in the right panels. Blue shaded areas depict the linescan recording site. The dark and light traces represent pre- and (late) post-tetanus data collected from the same cells depicted in Fig. 4e-4g. The hotspot area (pixels with >0.9 normalized pre-tetanus ΔF/F) expanded from a length of (B) during the pre-tetanus period to a combined length of (A)+(B)+(C) during the post-tetanus period, thus the (A)+(C) represents Δlengths.

d-f. The top panel displays the cumulative lengths of dendrites as a function of fluorescence level, while the bottom panel displays the corresponding Δlengths of the representative data shown in **a-c**. Cumulative lengths were obtained by summing up the dendritic length exhibiting the given fluorescence level, depicted as the dashed lines in **a** and the top panel of **d**. Δlengths were calculated by subtracting post-tetanus from pre-tetanus cumulative length, as indicated by the dashed line in **d**.

g-i. For all cells from all mice in each group, mean ± SEM of cumulative lengths (top panel) and Δlengths (bottom panel) as in **d-f**.

j-l. Mean ± SEM of Δlengths between pre- and early post-tetanus periods, as well as between pre- and late post-tetanus periods, as depicted in Fig. 4k-4m and **g-i**, respectively. The triangles in l indicate a decrease in Δlength from early post-tetanus period (solid triangle) to late post-tetanus period (open triangle).

m. Superimposed genotype Δlengths obtained from the bottom panel of **g-i**. The horizontal bar represents the median ± median absolute deviation of the distribution. Asterisks denote significance in distribution shift using Dunn & Sidák's approach (*, $p < 0.05$; **, $p < 0.01$; ***, $p < 0.001$) following Kruskal-Wallis test ($H[2] = 57.12$, $p < 0.001$, $n = 3929$).

n-o. Mean ± SEM of Δlengths, calculated from the off-hotspot (**n**) and hotspot (**o**) as grey shaded areas shown in **j**, with individual cell values alongside. (One-way ANOVA: off-hotspot, $F[2,153] = 9.77$, $p < 0.001$; hotspot, $F[2,153] = 21.37$, $p < 0.001$, $n = 624$).

Asterisks denote the significance levels of post hoc comparisons using Tukey's HSD (*, $p < 0.05$; **, $p < 0.01$; ***, $p < 0.001$).

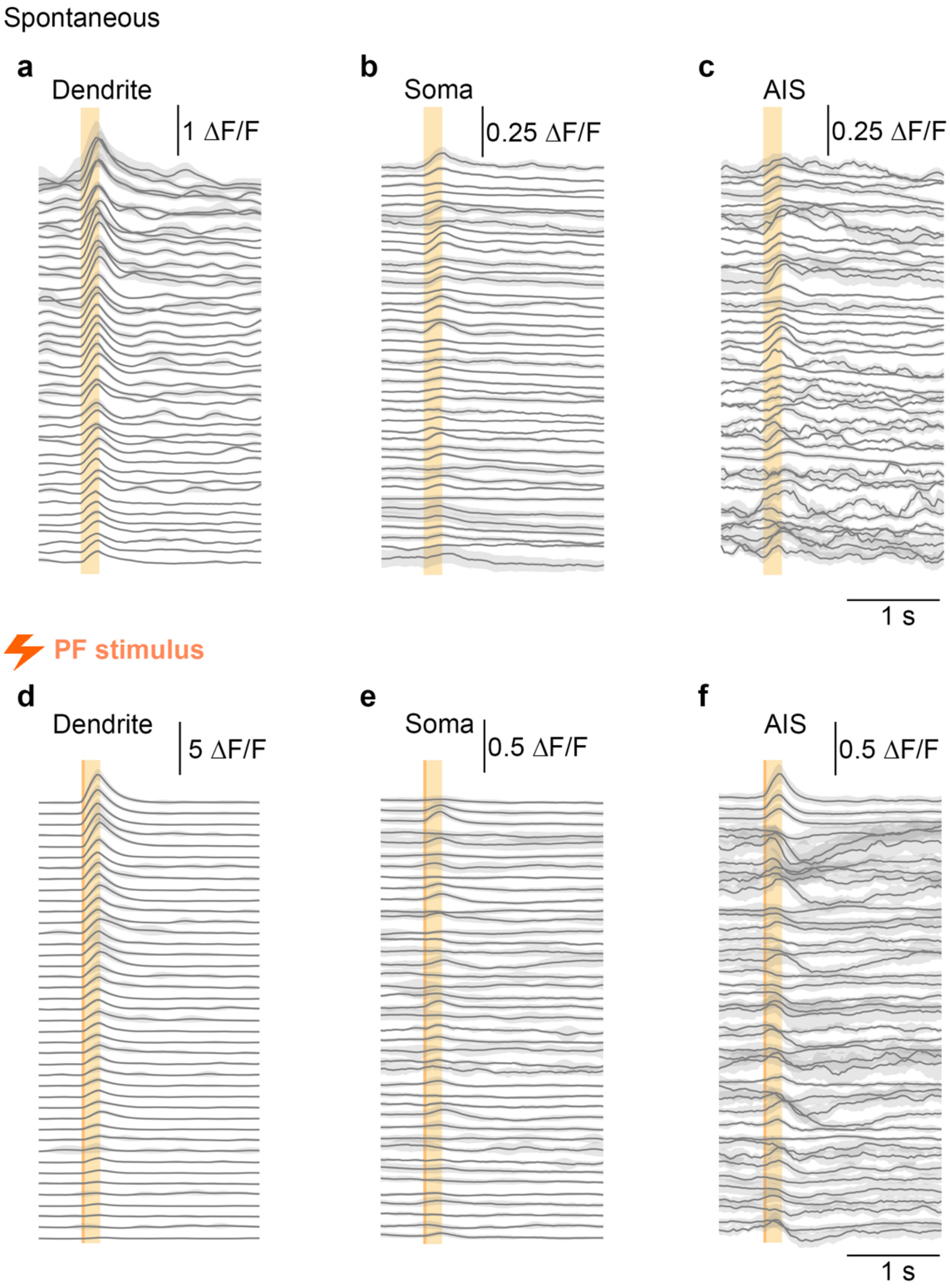

Supplementary Fig. 5 Most individual traces reveal time locked modulation of somatic and AIS calcium despite a saturated signal.

a-c. Spontaneous calcium transients (mean ± SEM) in dendrite (**a**), soma (**b**) and AIS (**c**) for each PC. Cells are sorted based on the amplitude of dendritic signals.

d-f. PF-evoked calcium transients (mean ± SEM) in dendrite (**d**), soma (**e**) and AIS (**f**) for each PC. Cells are sorted based on the amplitude of dendritic signals.

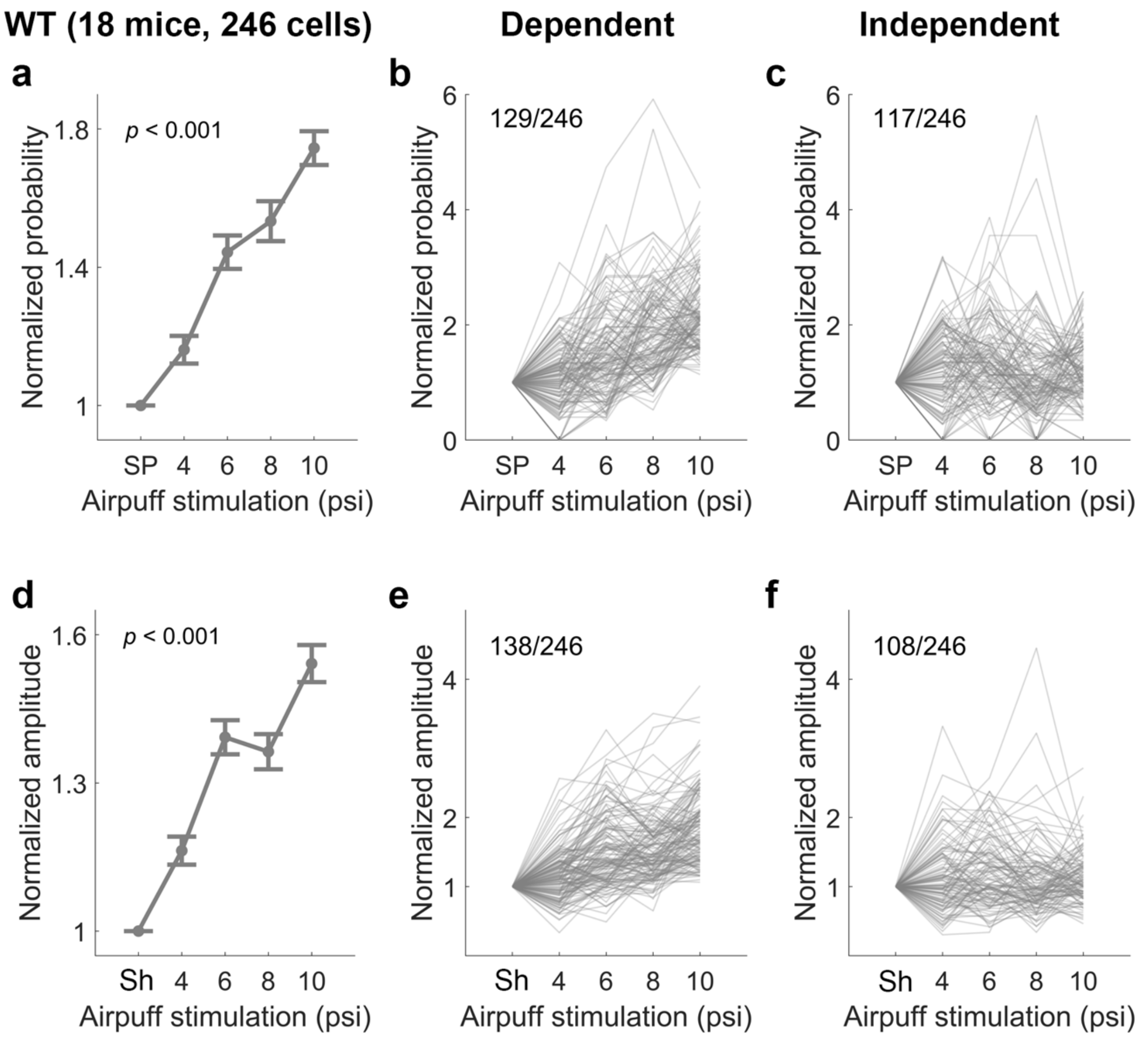

Supplementary Fig. 6 PCs show both linear stimulus intensity-dependent and flat stimulus intensity-independent responses to airpuff stimulation.

a. Calcium-event probability as depicted in Fig. 6d and 6f normalized by the spontaneous values of individual cells. (mean ± SEM). (One-way ANOVA: $F[4,1225] = 45.49$, $p < 0.001$, $n = 1230$).

b-c. Stimulus intensity-dependent (**b**) and stimulus intensity-independent cells (**c**) categorized according to the correlation coefficient of normalized probability. The labels on top of the plots indicate the number of designated cells out of the total number.

d. Calcium-event amplitude as depicted in Fig. 6g normalized by the maximum values of individual cells during shuffled 200ms time windows. (mean ± SEM). (One-way ANOVA: $F[4,1225] = 47.84$, $p < 0.001$, $n = 1230$).

e-f. Stimulus intensity-dependent (**e**) and stimulus intensity-independent cells (**f**) categorized according to the correlation coefficient of normalized probability. The labels on top of the plots indicate the number of designated cells out of the total number.

SP, spontaneous calcium events. Sh, shuffled traces.

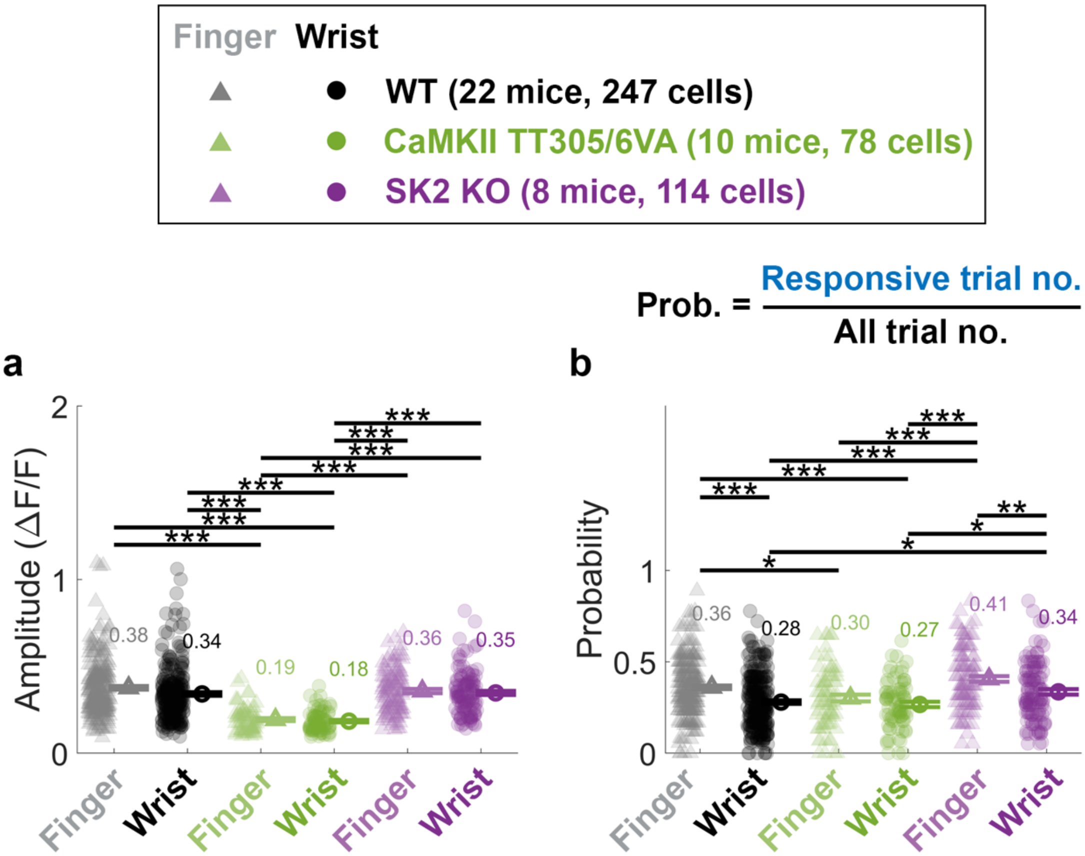

Supplementary Fig. 7 Amplitude and probability of pre-tetanus calcium events in response to airpuff stimulation to finger and wrist locations.

a. Mean ± SEM and individual cell values of the pre-tetanus amplitudes of calcium transients, calculated as the maximum value within the 0-200ms time window of individual trials. (One-way ANOVA: $F[5,855] = 37.19$, $p < 0.001$, $n = 878$).

b. Mean ± SEM and individual cell values of the pre-tetanus probability of detecting a calcium event within the defined time window of 0-200ms. (One-way ANOVA: $F[5,872] = 16.07$, $p < 0.001$, $n = 878$).

Numbers indicate the group mean values. Asterisks denote the significance levels of post hoc comparisons using Tukey's HSD (*, $p < 0.05$; **, $p < 0.01$; ***, $p < 0.001$).

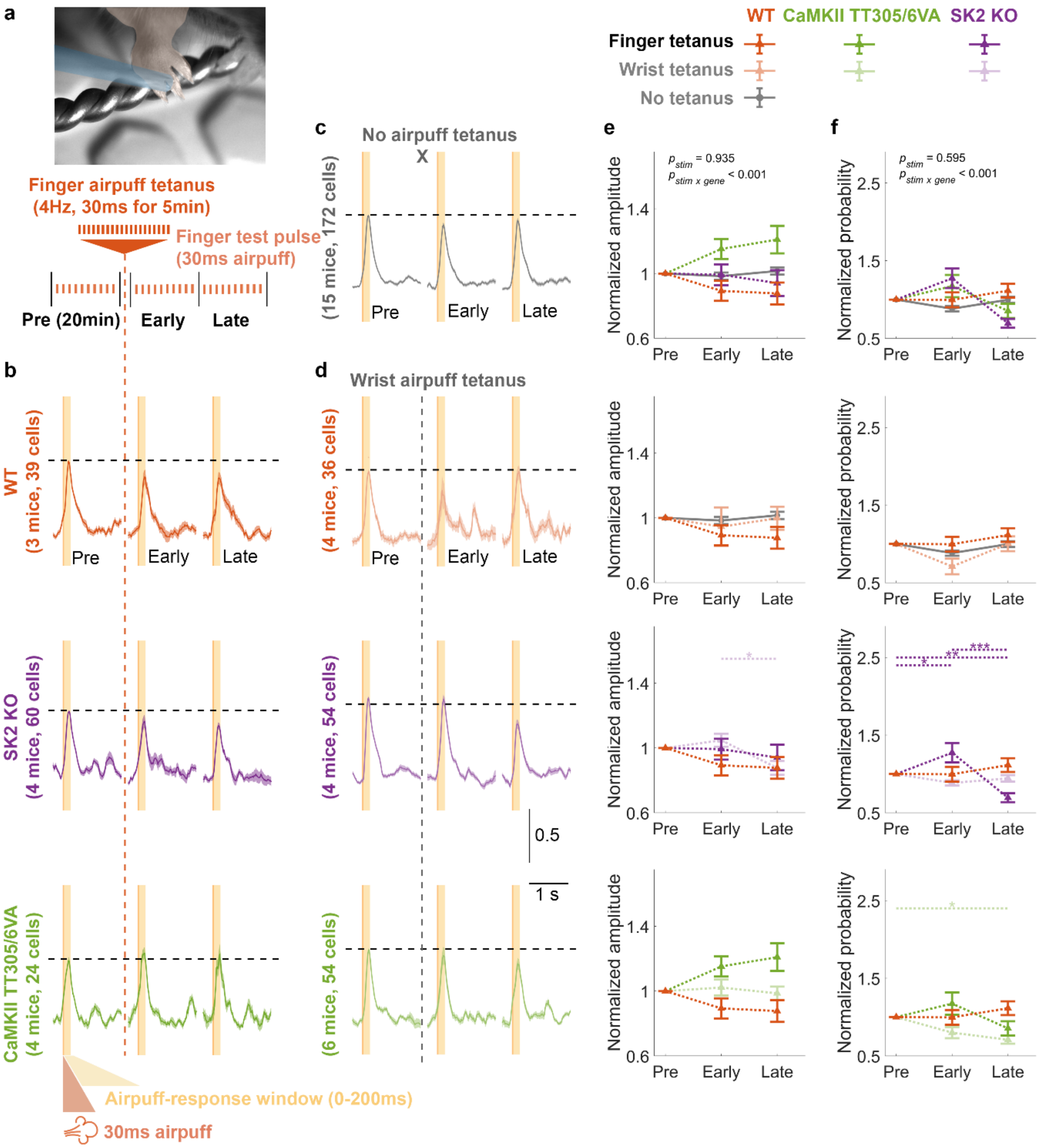

Supplementary Fig. 8 Airpuff finger stimulation does not induce detectable tactile-RF plasticity.

a. Schematic illustration of the experimental protocol. The recording began with a 20min baseline recording consisting of 10-12 trials, followed by a finger airpuff tetanus to potentiate the calcium response. The recording concluded with another 20min early and late post-tetanus recordings, each consisting of 10-12 trials. Post-tetanus trials began immediately (<2min) after cessation of the tetanus.

b. Normalized average calcium signals ± SEM across trials from different genotypes.

c. Normalized average calcium signals ± SEM across trials of the control condition in WTs where no tetanization protocol was applied.

d. Normalized average calcium signals ± SEM across trials from control conditions across genotypes where airpuff tetanization was applied to the wrist and thus was unmatched to the test location (finger). The signals shown in **b-d** are normalized by the average amplitude of pre-tetanus calcium transients (y-axis scale denotes normalized value units). Only trials with detected events within the response window (0-200ms) were included.
e. Mean ± SEM of the normalized calcium-event amplitudes, calculated as the maximum value within the 0-200ms time window of individual trials. The data were normalized by the pre-tetanus amplitude. (Two-way repeated measure ANOVA: $F_{stim}[2,1144] = 0.068$, $p = 0.935$; $F_{stim\ x\ gene}[24,1144] = 4.126$, $p < 0.001$, n = 2067).
f. Mean ± SEM of the normalized probability of detecting a calcium event within the 0-200ms time windows. The data were normalized by the pre-tetanus probability. (Two-way repeated ANOVA: $F_{stim}[2,1356] = 0.520$, $p = 0.595$; $F_{stim\ x\ gene}[24,1356] = 5.274$, $p < 0.001$, n = 2073).
Asterisks denote the significance levels of post hoc comparisons using Tukey's HSD (*, p < 0.05; **, p < 0.01; ***, p < 0.001).

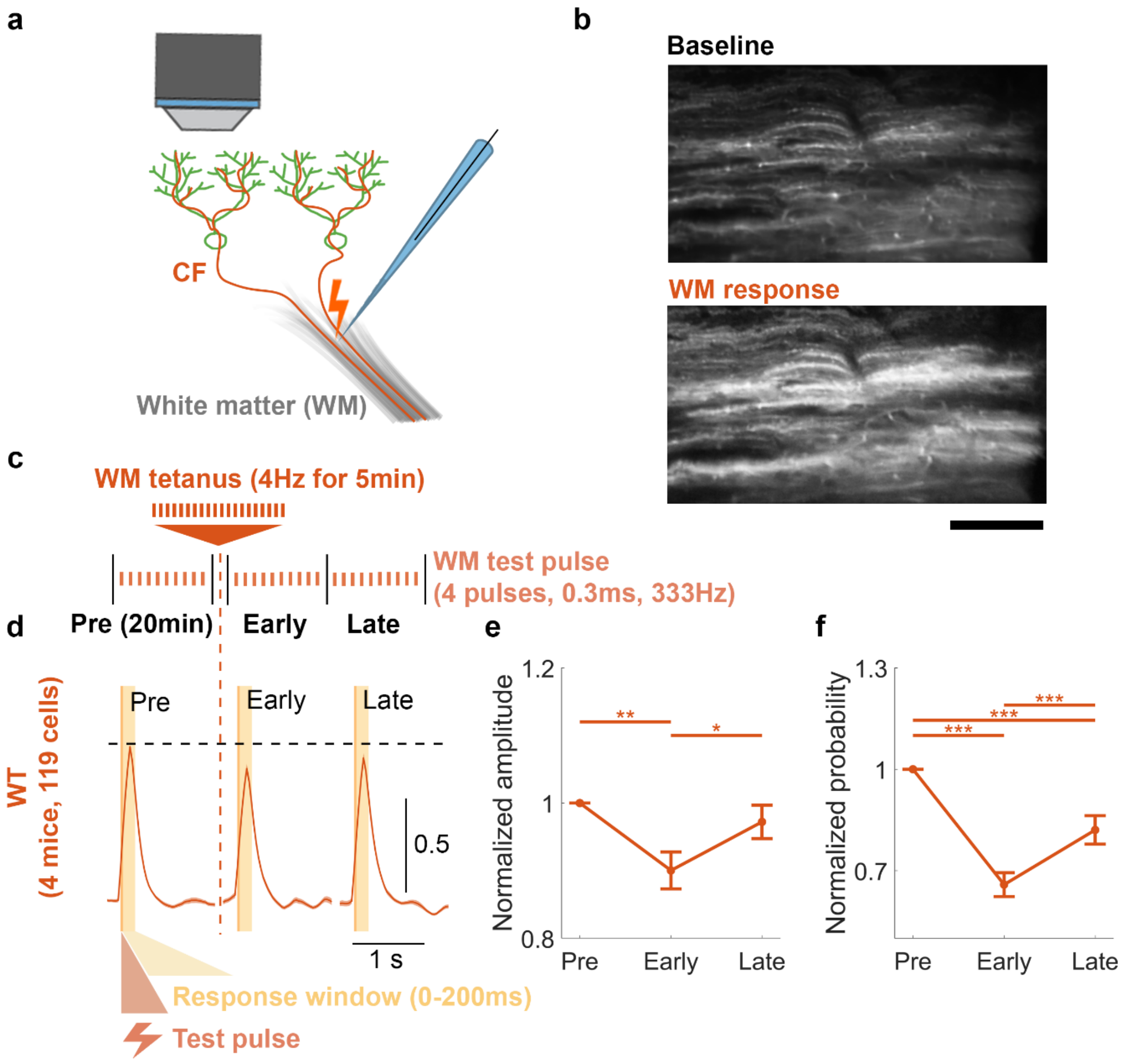

Supplementary Fig. 9 Applying the tactile tetanus protocol directly to upstream white matter tracts that include a strong CF component depresses the response in awake mice.
a. Schematic illustration of the experimental protocol *in vivo*.
b. Two-photon images demonstrating calcium signals in PCs during baseline and with application of the white matter stimulation test pulse. Scale bar is 100µm. Calcium imaging followed the same protocol as for PF-RF experiments (Fig. 1).
c. The recording began with a 20min baseline recording consisting of 10-12 trials, followed by a tetanization using the tactile tetanus protocol of 4Hz for 5min. The recording concluded with early and late post-tetanus recording periods, each consisting of 10-12 trials and lasting 20min. Post-tetanus trials began immediately (<2min) after cessation of the tetanus.
d. Normalized average calcium signals ± SEM for all trials. The signals are normalized by the average amplitude of pre-tetanus calcium transients (y-axis scale denotes normalized value units). Only trials with detected events within the response window (0-200ms) were included.
e. Mean ± SEM of the normalized calcium-event amplitudes, calculated as the maximum value within the 0-200ms time window of individual trials. The data were normalized by the pre-tetanus amplitude. (One-way ANOVA: $F[2,339] = 5.74$, $p = 0.004$, $n = 354$).

f. Mean ± SEM of the normalized probability of detecting a calcium event within the 0-200ms time windows. The data were normalized by the pre-tetanus probability. (One-way ANOVA: $F[2,351] = 28.98$, $p < 0.001$, $n = 357$).
Asterisks denote the significance levels of post hoc comparisons using Tukey's HSD (*, $p < 0.05$; **, $p < 0.01$; ***, $p < 0.001$).

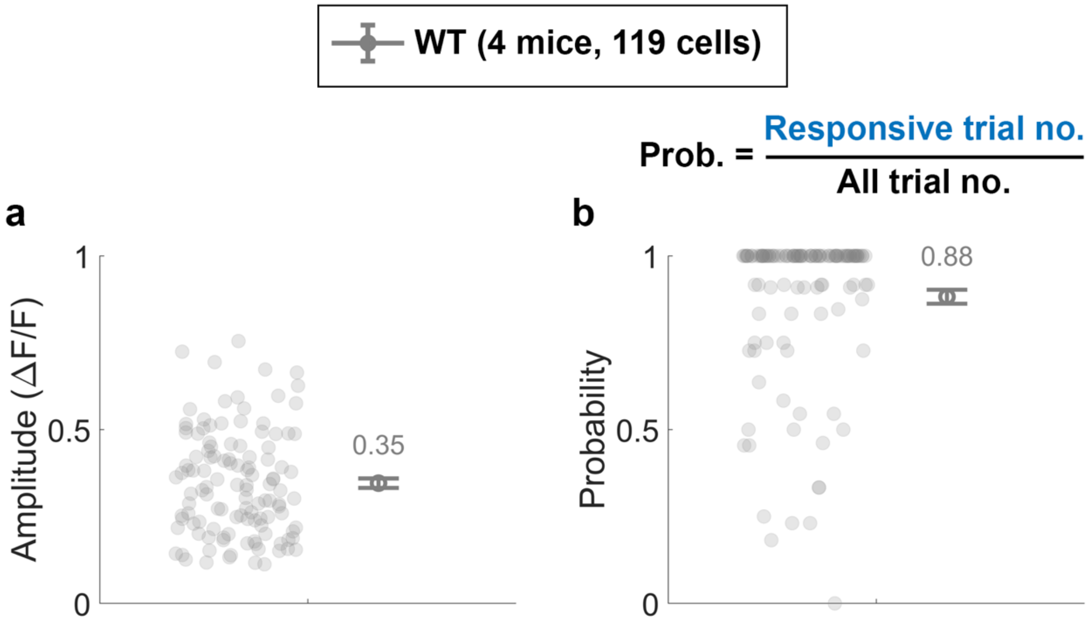

Supplementary Fig. 10 Amplitude and probability of pre-tetanus calcium events in response to white matter stimulation.
a. Mean ± SEM (n = 118) of the pre-tetanus amplitudes of calcium transients for responsive trials.
b. Mean ± SEM (n = 119) of the pre-tetanus probability of detecting a calcium event within the defined time window of 0-200ms.
Numbers indicate the group mean values.
