# Supplementary material for: Intrinsic and synaptic determinants of receptive field plasticity in Purkinje cells of the mouse cerebellum
Source: Nat Commun. 2024 May 31;15:4645. doi: 10.1038/s41467-024-48373-3 (PMC11143328; doi:10.1038/s41467-024-48373-3)
Supplement: Supplementary file 3 — Reporting Summary [file 41467_2024_48373_MOESM3_ESM.pdf]

nature portfolio

Corresponding author(s): Christian Hansel

Last updated by author(s): 04/02/2024

Reporting Summary

Nature Portfolio wishes to improve the reproducibility of the work that we publish. This form provides structure for consistency and transparency in reporting. For further information on Nature Portfolio policies, see our Editorial Policies and the Editorial Policy Checklist.

Statistics

For all statistical analyses, confirm that the following items are present in the figure legend, table legend, main text, or Methods section.

n/a	Confirmed	
☐	☒	The exact sample size (*n*) for each experimental group/condition, given as a discrete number and unit of measurement
☐	☒	A statement on whether measurements were taken from distinct samples or whether the same sample was measured repeatedly
☐	☒	The statistical test(s) used AND whether they are one- or two-sided<br>*Only common tests should be described solely by name; describe more complex techniques in the Methods section.*
☐	☒	A description of all covariates tested
☐	☒	A description of any assumptions or corrections, such as tests of normality and adjustment for multiple comparisons
☐	☒	A full description of the statistical parameters including central tendency (e.g. means) or other basic estimates (e.g. regression coefficient) AND variation (e.g. standard deviation) or associated estimates of uncertainty (e.g. confidence intervals)
☐	☒	For null hypothesis testing, the test statistic (e.g. *F*, *t*, *r*) with confidence intervals, effect sizes, degrees of freedom and *P* value noted<br>*Give P values as exact values whenever suitable.*
☒	☐	For Bayesian analysis, information on the choice of priors and Markov chain Monte Carlo settings
☐	☒	For hierarchical and complex designs, identification of the appropriate level for tests and full reporting of outcomes
☐	☒	Estimates of effect sizes (e.g. Cohen's *d*, Pearson's *r*), indicating how they were calculated

Our web collection on statistics for biologists contains articles on many of the points above.

Software and code

Policy information about availability of computer code

Data collection	Two-photon image was recorded using Scanbox software (Scanbox, Los Angeles, CA) followed by image processing conducted using custom-made scripts in MATLAB R2017b (MathWorks, Natick, MA, USA).
Data analysis	The code is written in MATLAB R2021b and publicly available from GitHub: https://github.com/tingfenglin-ac/CerebellumRFplasticity

For manuscripts utilizing custom algorithms or software that are central to the research but not yet described in published literature, software must be made available to editors and reviewers. We strongly encourage code deposition in a community repository (e.g. GitHub). See the Nature Portfolio guidelines for submitting code & software for further information.

Data

Policy information about availability of data

All manuscripts must include a data availability statement. This statement should provide the following information, where applicable:

- Accession codes, unique identifiers, or web links for publicly available datasets
- A description of any restrictions on data availability
- For clinical datasets or third party data, please ensure that the statement adheres to our policy

Data presented in this study are saved as MAT-file (version 7.0) and publicly available from Mendeley Data: https://doi.org/10.5281/zenodo.8045156

Research involving human participants, their data, or biological material

Policy information about studies with human participants or human data. See also policy information about sex, gender (identity/presentation), and sexual orientation and race, ethnicity and racism.

Reporting on sex and gender	N/A
Reporting on race, ethnicity, or other socially relevant groupings	N/A
Population characteristics	N/A
Recruitment	N/A
Ethics oversight	N/A

Note that full information on the approval of the study protocol must also be provided in the manuscript.

Field-specific reporting

Please select the one below that is the best fit for your research. If you are not sure, read the appropriate sections before making your selection.

☒ Life sciences ☐ Behavioural & social sciences ☐ Ecological, evolutionary & environmental sciences

For a reference copy of the document with all sections, see nature.com/documents/nr-reporting-summary-flat.pdf

Life sciences study design

All studies must disclose on these points even when the disclosure is negative.

Sample size	No statistical methods were used to predetermine sample sizes, however, the sample sizes used in this study are similar to those used in previous publications in the field applying the same techniques and/or use of multiple genotypes (Simmons et al., 2022, Busch and Hansel 2023, Busch et al., 2023). The parallel fiber (PF) stimulus intensity experiments were conducted in 11 wild-type, 10 SK2-KO, and 8 CaMKII-TT305/6VA mice. PF receptive field (RF) plasticity experiments were conducted in 12 wild-type, 7 SK2-KO, and 7 CaMKII-TT305/6VA mice, all largely the same animals as used for the stimulus intensity experiments. Spatial dendrite analysis was performed on a subset of PF-RF experimental recordings. Simultaneous recordings of dendrite, soma, and axon initial segment (AIS) were conducted in 5 wild-type mice. The tactile-RF plasticity experiments (finger vs wrist tetanization combined) were conducted in 14 wild-type, 8 SK2-KO, and 10 CaMKII-TT305/6VA mice. Climbing fiber (CF) RF plasticity experiments were conducted in 4 wild-type mice.
Data exclusions	No data were excluded.
Replication	Data was obtained for each genotype and experimental approach from >3 animal cohorts across breeding pairs and litters and were tested independently by two authors. Trends in the data show no dependence on date, experimenter, or animal cohort.
Randomization	The mice selected for each experiment were randomly chosen from each mouse line.
Blinding	Complete blinding was not logistically possible at the experimental stage, but most experiments required two experimenters to participate and the one overseeing the procedure was always blind to genotype. All data analysis pipelines treat the datasets equally such that analyses and statistics were effectively blinded.

Reporting for specific materials, systems and methods

We require information from authors about some types of materials, experimental systems and methods used in many studies. Here, indicate whether each material, system or method listed is relevant to your study. If you are not sure if a list item applies to your research, read the appropriate section before selecting a response.

Materials & experimental systems

n/a	Involved in the study
☒	☐ Antibodies
☒	☐ Eukaryotic cell lines
☒	☐ Palaeontology and archaeology
☐	☒ Animals and other organisms
☒	☐ Clinical data
☒	☐ Dual use research of concern
☒	☐ Plants

Methods

n/a	Involved in the study
☒	☐ ChIP-seq
☒	☐ Flow cytometry
☒	☐ MRI-based neuroimaging

Animals and other research organisms

Policy information about studies involving animals; ARRIVE guidelines recommended for reporting animal research, and Sex and Gender in Research

Laboratory animals	All mice were bred on a C57BL/6J background (The Jackson Laboratory, Bar Harbor, ME, USA) and maintained in a standard 12hr light/dark cycle at 72degrees Fahrenheit and 47% humidity. All genotypes were regularly outcrossed with commercial wild-type C57BL/6J mice. P80-P120 adult mice of all genotypes were selected for the two-photon recordings performed in this study, and the necessary surgeries were conducted 2-3 weeks prior to the recording.<br>SK2 knockout mice (SK2-KO) were generated by crossing transgenic mice expressing CRE recombinase under the Purkinje cell (PC)-specific promoter Pcp2/L7 (JAX#010536) with transgenic mice with LOXP sites flanking the Kcnn2 locus encoding SK2 protein (see the previous report1 for detail). Mice with L7-Cre and homozygous SK2-LoxP were used for the experimental mutant group. CaMKII TT305/6VA genetically modified mice, which carry a global mutaiton, were obtained from Dr. Ype Elgersma (Erasmus Medical Center, The Netherlands). Wild-type mice used in the current studies were collected from the litter mates of both mutants confirmed by genotyping.
Wild animals	No wild animals were used in this study.
Reporting on sex	Animals of both sexes were used in all experiments. No sex differences were observed in the reported measures.
Field-collected samples	No field collected samples were used in this study.
Ethics oversight	All animal experiments were approved and conducted in accordance with the regulations and guidelines of the Institutional Animal Care and Use Committee of the University of Chicago (IACUC 72496).

Note that full information on the approval of the study protocol must also be provided in the manuscript.

Plants

Seed stocks	N/A
Novel plant genotypes	N/A
Authentication	N/A
